# Genome-wide investigation of the GRAS transcription factor family in foxtail millet (*Setaria italica* L.)

**DOI:** 10.1186/s12870-021-03277-y

**Published:** 2021-11-03

**Authors:** Yu Fan, Xiaobao Wei, Dili Lai, Hao Yang, Liang Feng, Long Li, Kexin Niu, Long Chen, Dabing Xiang, Jingjun Ruan, Jun Yan, Jianping Cheng

**Affiliations:** 1 College of Agriculture, Guizhou University, Guiyang, 550025 People’s Republic of China; 2 School of Food and Biological engineering, Chengdu University, Chengdu, 610106 People’s Republic of China; 3 Guizhou provincial Center For Disease Control And Prevention, Guiyang, 550025 People’s Republic of China; 4 Chengdu Institute of Food Inspection, Chengdu, 610030 People’s Republic of China; 5 Henan university of technology, Zhengzhou, 450001 People’s Republic of China; 6 Department of Nursing, Sichuan Tianyi College, Mianzhu, 618200 People’s Republic of China

**Keywords:** *Setaria italica*, *GRAS* gene family, Genome-wide analysis, DELLA protein, Fruit development, Abiotic stress

## Abstract

**Background:**

GRAS transcription factors perform indispensable functions in various biological processes, such as plant growth, fruit development, and biotic and abiotic stress responses. The development of whole-genome sequencing has allowed the *GRAS* gene family to be identified and characterized in many species. However, thorough in-depth identification or systematic analysis of *GRAS* family genes in foxtail millet has not been conducted.

**Results:**

In this study, 57 *GRAS* genes of foxtail millet (*SiGRASs*) were identified and renamed according to the chromosomal distribution of the *SiGRAS* genes. Based on the number of conserved domains and gene structure, the *SiGRAS* genes were divided into 13 subfamilies via phylogenetic tree analysis. The *GRAS* genes were unevenly distributed on nine chromosomes, and members of the same subfamily had similar gene structures and motif compositions. Genetic structure analysis showed that most *SiGRAS* genes lacked introns. Some *SiGRAS* genes were derived from gene duplication events, and segmental duplications may have contributed more to *GRAS* gene family expansion than tandem duplications. Quantitative polymerase chain reaction showed significant differences in the expression of *SiGRAS* genes in different tissues and stages of fruits development, which indicated the complexity of the physiological functions of *SiGRAS*. In addition, exogenous paclobutrazol treatment significantly altered the transcription levels of DELLA subfamily members, downregulated the gibberellin content, and decreased the plant height of foxtail millet, while it increased the fruit weight. In addition, *SiGRAS13* and *SiGRAS25* may have the potential for genetic improvement and functional gene research in foxtail millet.

**Conclusions:**

Collectively, this study will be helpful for further analysing the biological function of *SiGRAS*. Our results may contribute to improving the genetic breeding of foxtail millet.

**Supplementary Information:**

The online version contains supplementary material available at 10.1186/s12870-021-03277-y.

## Background

GRAS proteins belong to a family of plant-specific transcription factors and have been widely reported to exist in higher plants [1]. The *GRAS* family name was based on the first three members: gibberellic acid insensitive (*GAI*) [2], repressor of GA1-3 mutant (*RGA*) [3], and scarecrow (*SCR*) [4]. GRAS proteins usually contain 400–770 amino acid residues [5]. The N-terminal of GRAS proteins constituted an intrinsically disordered region (IDR) peculiar to plants. This domain is highly variable and can switch between an irregular and regular structures, which may be used as a molecular bait to bind to different target proteins. GRAS proteins involved in different signal transduction pathways and a variety of functions, thereby determining the specificity of their functions [5]. Differentiation at the N-terminal further leads to the diversification of GRAS proteins, for example, the DELLA proteins are characterized by the DELLA domain at the N-terminal [6]. Meanwhile, the C-terminal sequence of GRAS transcription factors is relatively conserved, has specific transcription regulatory domains that bind to DNA or other proteins, and can recognize a variety of different target receptors. Thus, the C-terminal sequence participates in a variety of signal transduction regulated transcription processes [2–4]. The C-terminal of GRAS protein contains five highly conserved domains:leucine-heptad repeat I (LHR I), Val-His-Ile-Ile-Asp (VHIID), leucine-heptad repeat II (LHR II), Pro-Phe-Tyr-Arg-Glu (PFYRE), and Ser-Ala-Trp (SAW) [7]. Of these domains, the conserved structural domains of GRAS proteins, the VHIID region can be used as the core region and exists in almost all GRAS proteins: V, I, H, and D represent valine, isoleucine, histidine, and aspartic acid respectively [7, 8]. The two leucine-rich regions do not have the phenomenon of seven duplicated leucine residues that form the leucine zipper [5, 9, 10]. The posterior segment of the LHR II domain contains Leucine-X-X-Leucine-Leucine (LXXLL, where X stands for any amino acid) structure and is conserved in most GRAS proteins [11]. Although the functions of the PFYRE and SAW regions have not yet been elucidated, their high conservatism is suggested to be closely related to GRAS protein function [12]. The PFYRE domain shows collinearity and high similarity in all proteins, usually consisting of three components: proline residues (P), phenylalanine and tyrosine residues (FY), arginine and glutamic acid residues (RE), and other structural domains [11, 13, 14]. The SAW motif comprises three consecutive parts near the C terminal, namely WX7G (X7 represents any seven amino acids), L-W, and SAW. These conserved elements play an important role in maintaining the integrity of the GRAS domain [13, 14].

Tian et al. [15] have systematically analyzed the GRAS family genes in *Arabidopsis thaliana* and rice. According to phylogenetic analysis, 57 *GRAS* genes in rice and 33 in *Arabidopsis* were divided into eight branches (DELLA, PAT1, LISCL, SCL3, SCR, LS, SHR, and HAM), which were named according to the representative genes in these subfamilies. In 2017, Cenci et al. [16] analyzed *GRAS* gene family members in different angiosperms, providing a clear basis for their classification. In angiosperms, the *GRAS* gene family not only includes the homologous members of the eight *Arabidopsis GRAS* subfamilies but also includes those of many other subfamilies (e.g., the NSF1, NSP2, and DLT subfamilies). GRAS proteins not only different in terms of their structure but also play a variety of physiological functions in plant growth [17]. For example, SCR is involved in cell division in the cortex and endodermis of roots in *Arabidopsis*, while SHR regulates the asymmetric division of roots by activating SCR proteins [4, 18, 19]. The interaction between SHR-SCR heterodimer and BIRD/IDD transcription factors was a typical example of target-effector proteins recognition by GRAS proteins [20]. *NSP1* and *NSP2* can form homodimer or heterodimer, which are necessary for nodular development in legumes [21]. *AtLAS* regulates lateral bud formation during vegetative growth in *A. thaliana* [22]. *LeLs* (*Lycopersicon esculentum* lateral suppressor), a member of the AtLAS subfamily in tomato, is mainly responsible for stimulating lateral meristem development during the vegetative growth stage to promote lateral bud formation [23, 24]. *OsMOC1*, a homologous protein of the AtLAS subfamily in rice, plays an important role in lateral meristem development and tiller bud formation [25]. *PAT1* and *SCL13* of the PAT1 branch act as intermediates in the photosensitive pigment signaling pathway [24, 26]. Furthermore, AtSCL3 protein is mainly expressed in the endodermis of roots and located in the nucleus of root cells as a positive regulator of gibberellin (GA) signal transduction [18, 19]. AtSCL3 and DELLA proteins play antagonistic roles in regulating the downstream GA response and GA homeostasis. DELLA protein exists in the GA signal transduction pathway as a negative regulator. When the GA signal recognition region on DELLA protein receives the signal, the protein degrades rapidly in the nucleus, and the plant shows a normal GA response program. Meanwhile, DELLA-gene-mutant plants will show dwarfization and a GA-insensitive phenotype [27]. In addition, DELLA protein can regulate reproductive organ development and promote fruit growth of *Arabidopsis* by affecting the fertilization process [28]. *PhHAM* of petunia is mainly expressed in the lateral organ primordium and the vascular tissue of the stem primordium. *PhHAM* acts on adjacent tissues in a noncellular autonomous manner to maintain the activity of the meristem of the shoot tip. The number of leaves in the petunia mutant (*ham*) is less than that in the wild type. Furthermore, the stem apical meristem loses its undifferentiated characteristics and forms a differentiated epidermis through the trichome, thereby preventing further organ formation [29]. The LISCL protein is regarded as the transcriptional activator of some meiosis-related genes, regulating anther microspore formation [30]. *OsDLT* regulates shoot and primary root development in rice, showing a dwarf phenomenon that is insensitive to brassinolide [31]. In addition, *GRAS* family members are also involved in plant responses to multiple environmental stresses. For example, the *PeSCL7* in poplar can be induced by drought and high-salt stresses [32]. Salt stress, cold stress, and osmotic stress can reduce active GA content in plants, which can cause DELLA protein accumulation, inhibit plant growth, and enhance resistance to environmental stresses [33, 34].

Foxtail millet (*Setaria italica*) is an annual diploid C4 crop belonging to the Gramineae family. It has a short growth cycle and is warming-loving and reduced-sunshine and drought-tolerant [35]. Foxtail millet originated in China and is widely cultivated in arid and semiarid regions of the world as food and fodder [36]. The *GRAS* gene family was identified in model organisms *A. thaliana* and rice [15, 17], and it has been gradually identified and analyzed at the genome-wide level in more and more species, such as *Solanum lycopersicum* [37], *Vitis vinifera* [38], *Malus domestica* [39], *Zea mays* [40], *Gossypium hirsutum* [41], *Fagopyrum tataricum* [42], and *Sorghum bicolor* [43] and others. As a typical C4 plant, foxtail millet has not been studied for the identification and candidate gene screening of the *GRAS* family. In this study, 57 *GRAS* genes in foxtail millet were analyzed, and they were divided into 13 groups. In addition, the exon-intron structure, motif composition, gene replication, chromosome distribution, and phylogenetic relationships were further analyzed. The expression of *SiGRAS* family members under different biological processes and abiotic stresses was also assessed.

## Results

### Identification of *GRAS* genes in *S. italica*

In this study, two BLAST methods were used to identify all possible *GRAS* members in the *S. italica* genome (Table S1). According to their location on chromosomes, the *SiGRAS* members were renamed *SiGRAS01* to *SiGRAS57*. The basic characteristics that were analyzed, which included molecular weight (MW), isoelectric point (pI), coding sequence length (CDS), and subcellular localization (http://cello.life. nctu.edu.tw/).

Of the 57 SiGRAS proteins, SiGRAS19 was the smallest with 248 amino acids, and the largest was SiGRAS26 with 912 amino acids. The molecular masses of the proteins ranged from27.70 kDa (SiGRAS20) to 100.09 kDa (SiGRAS26) and the pI ranged from 4.85 (SiGRAS16) to 9.53 (SiGRAS03), with a mean of 6.35. In the predicted subcellular localization results, 26 SiGRAS proteins were located in the nucleus, 16 were located in the cytoplasm, 13 were located in the chloroplasts, 1 was located in the chloroplasts, and 1 was located in the mitochondria (Table S1). The number of GRAS genes in *S. italica* was higher than that in *A. thaliana* (32) [15], *Cucumis sativus* (37) [44], *Vitis vinifera* (52) [38], and Tartary buckwheat (47) [42], and lower than that in *Sorghum bicolor* (81 ) [43]*, Populus trichocarpa* (102) [45], and *Malus* x *domestica* (127) [39].

### Multiple sequence alignment, phylogenetic analysis, and classification of *SiGRAS* genes

**Fig. 1:**
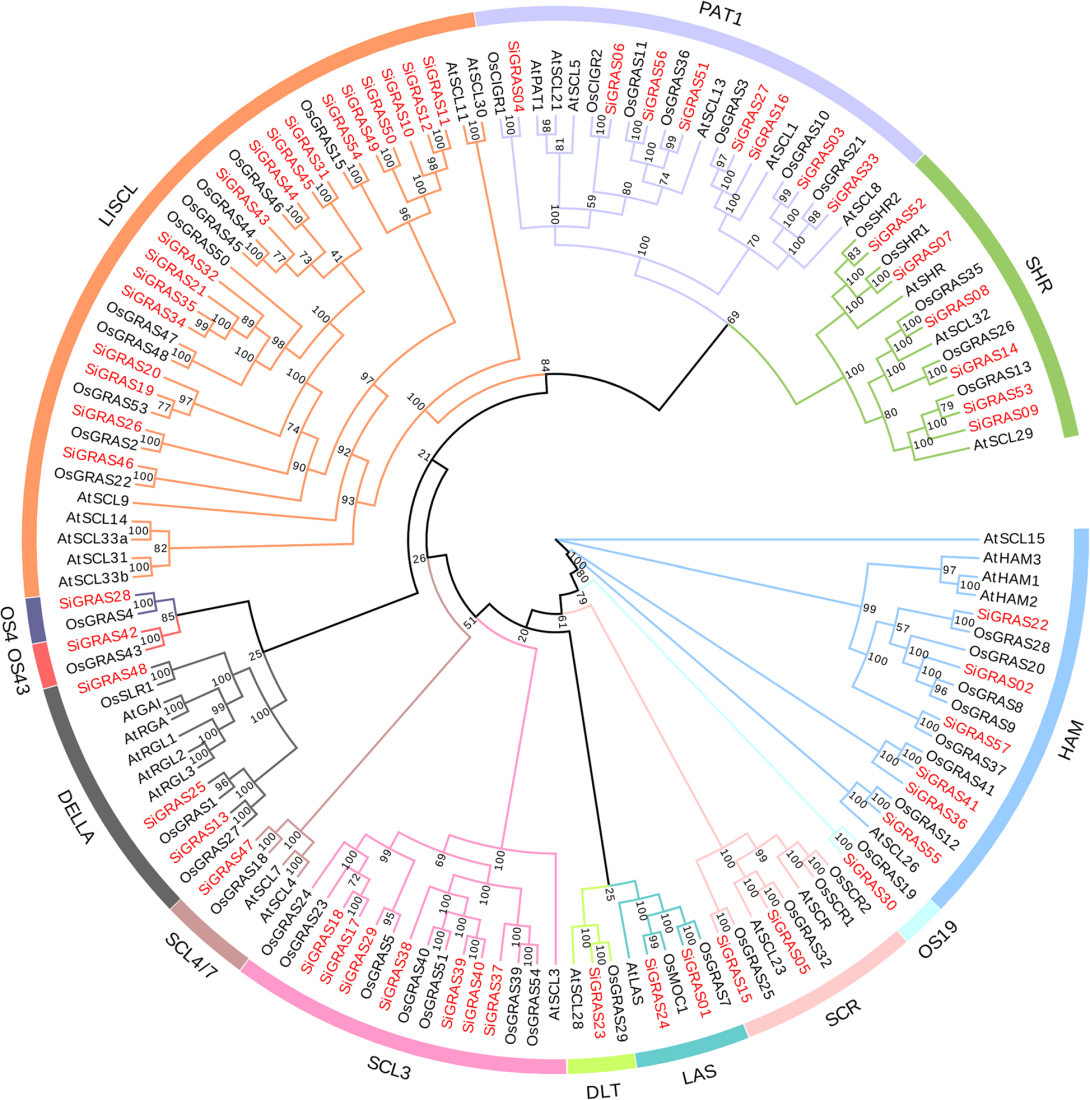
Unrooted phylogenetic tree representing relationships among GRAS domains of *S. italica*, *Arabidopsis*, and rice. The phylogenetic trees were derived using the NJ method in MEGA7.0. The tree shows the 13 phylogenetic subfamilies marked with the red font on a white background. GRAS proteins from *Arabidopsis* and *O. sativa* have the prefix ‘At’ and ‘Os’, respectively

To investigate the phylogenetic relationship of GRAS proteins in the foxtail millet, we constructed a phylogenetic tree based on the amino acid sequences of the 57 identified *SiGRAS*, 33 AtGRAS, and 50 OsGRAS proteins (Fig. 1, Tables S1 and S2). According to the previously proposed classification method and topological structure proposed by Cenci and Rouard [16], the 140 GRAS proteins in the phylogenetic tree were divided into 13 main clades. This finding was consistent with the previous classification of the GRAS taxonomic subfamilies in angiosperms. These data indicate that these proteins had no loss during the evolution of *S. italica*. These subfamilies of GRAS proteins widely existing in different angiosperms may play a basic role in plant development and evolution, similar to those recently reported in other plant species, including *Amborella trichopoda*, *Phoenix dactylifera*, *V. vinifera*, *Musa acuminata*, *O. sativa*, *A. thaliana*, *Theobroma cacao* and *Coffea canephora* [16]. Among the 13 subfamilies, LISCL had the most members (18 SiGRAS proteins), and DLT (SiGRAS23), SCL4/7(SiGRAS47), OS19(SiGRAS30), OS4(SiGRAS28), and OS43(SiGRAS42) had the fewest (only one SiGRAS protein) (Fig. 1, Tables S1 and S2). There were eight, seven, six, six, three, two, and two SiGRAS proteins in the PAT1, SCL3, SHR, HAM, DELLA, SCR, and LAS subfamilies, respectively. Some SiGRAS proteins were tightly grouped with the AtGRAS and OsGRAS proteins (bootstrap support ≥70). These proteins may be orthologous to AtGRASs or OsGRASs, and may have similar physiological functions.

These GRAS proteins of *Arabidopsis* and rice, which have high homology and similar protein structures to SiGRASs, were selected for multiple sequence alignment. Furthermore, the LHR I, VHIID, LHR II, PFYRE, and SAW domains of these GRAS proteins were further compared. As shown in Fig. S1, the VHIID domain is considered to be the core region, which contained a characteristic amino acid sequence. Although its amino acid structure was highly similar in different plant species, this region was not completely conserved. Further, the His and Asp amino acid residues in the domain were more conserved, and these residues may be necessary for the function of GRAS proteins in different subfamilies [15–17]. Similar to the GRAS proteins of sorghum [43] and rice [15], the N-terminal of SiGRAS proteins contains a highly disordered region, and shows certain similarities among different subfamilies [5].

### Conserved motifs, gene structures, and cis-acting elements analysis of *SiGRAS* genes

**Fig. 2:**
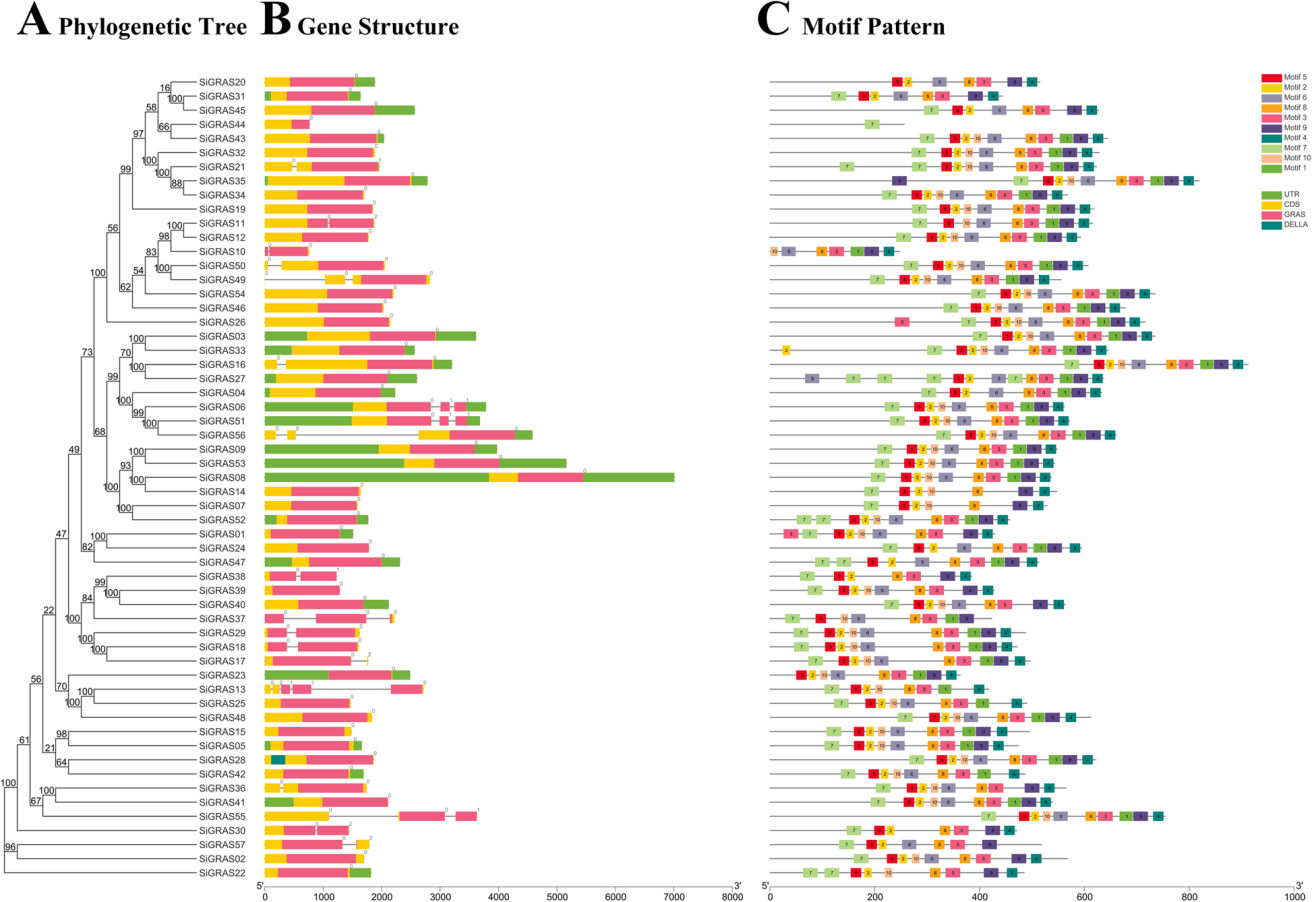
Phylogenetic relationship, gene-structure analysis, and motif distributions of *S. italica GRAS* genes. **A** Phylogenetic tree was constructed by the NJ method with 1000 replicates on each node. **B** Exons and introns are indicated by rectangles and gray lines, respectively. These numbers are generated based on the “phase” of the annotated file, which is about the different phases of CDS in the gene. Meanwhile, “phase” is defined as “0”, “1”, and “2”. **C** Amino acid motifs in the *S. italica* GRAS proteins (1–10) are represented by colored boxes. The black lines indicate relative protein lengths

By comparing the genomic DNA sequences of SiGRAS genes, we obtained the intron and exon structures of SiGRAS genes to further understand the structural composition of these genes (Fig. 2, Tables S1 and S3). A comparison of the localization and number of the exon-intron structures revealed that the 57 SiGRAS genes had different numbers of exons, varying from 1 to 5 (Fig. 2A, B). The 57 *SiGRAS* genes all contained the GRAS domain, and most of them (37, ~ 64.91%) contained no introns; 13 *SiGRAS* genes contained one intron; *SiGRAS04, SiGRAS12, SiGRAS16, SiGRAS27, SiGRAS38*, and *SiGRAS42* contained two introns; and *SiGRAS18* had four introns, which was the highest number of introns. The 37 genes without introns were distributed across the other 12 subfamilies, except for the Os43 subfamily, and were mainly part of the LISCL subfamily. In general, members of the same subfamily had similar gene structures. Members of the DELLA, DLT, HAM, LAS, OS19, OS4, SCL4/7, SCR, and SHR subfamilies contained no introns or one intron. Further analyses indicated that the SCL3 subfamily was the most diverse in terms of the number of introns.

To further study the characteristic regions of the *SiGRAS* proteins, their motifs were analyzed using an online MEME. A total of 10 distinct conserved motifs (named motifs 1–10) were found (Fig. 2C, Table S3). As exhibited in Fig. 2C, motif 8, 3, 9, and 4 were widely distributed in the *SiGRAS* family, except for *SiGRAS41*. *SiGRAS* members of the same subfamily usually shared a similar motif composition. For example, the DELLA subfamily contained motifs 7, 5, 2, 10, 6, 8, 3, 1, 9, and 4; the LISCL subfamily contained motifs 6, 8, 3, 9, and 4; and the HAM subfamily contained motifs 5, 2, 8, 3, and 9. Some motifs were only distributed in specific locations of the pattern. For example, the motif 7 (50, ~ 87.72%) was always distributed at the start of the pattern, and motif 4 (55, ~ 96.49%) was almost always at the end of the pattern. The functions of most of these conserved motifs remain to be elucidated.

The cis-acting elements in the promoter regions (2000 bp) of 57 *SiGRAS* genes were further investigated. A total of 110 cis-regulatory elements were identified (Table S4), which could be divided into seven categories: development-related, environmental stress-related, hormone-responsive, light-responsive, promoter-related, site binding-related, and others. Among them, the light-response elements accounted for a large proportion, including 30 cis-regulatory factors. The promoter related element (CAAT-box, TATA-box elements) and other elements (MYB, MYC, and the unnamed 4 elements) in the promoter region were identified in all *SiGRAS* genes (57 members). There were ten hormone-responsive elements in the 57 *SiGRAS* genes of the foxtail millet, which covered most plant hormones, including abscisic acid responsive (ABRE), auxin responsive (AuxRR-core, TGA-element), gibberellin-responsive (GARE-motif, P-box, TATC-box), jasmonic acid-responsive (CGTCA-motif, TGACG-motif), and salicylic acid-responsive (TCA-element) elements. In addition, cis-regulatory elements related to low-temperature, anoxia, drought, anaerobic conditions, other defenses, and stress responses were found in *SiGRAS* genes. Nearly 85% of *SiGRAS* genes contained anaerobic induction-responsive elements, light-responsive elements, abscisic acid-responsive elements, and MeJA responsive-responsive elements; whereas only approximately 28% of GRAS genes contained GA--responsive and auxin-responsive elements. Some cis-acting elements may regulate the expression of different tissues (seed, root, endosperm, palisade mesophyll, and meristem) during development. It can be inferred that *SiGRAS* genes can not only participate in the tissue development process but also respond to a variety of abiotic stresses.

### Chromosomal spread and gene duplication in *SiGRAS* genes

**Fig. 3:**
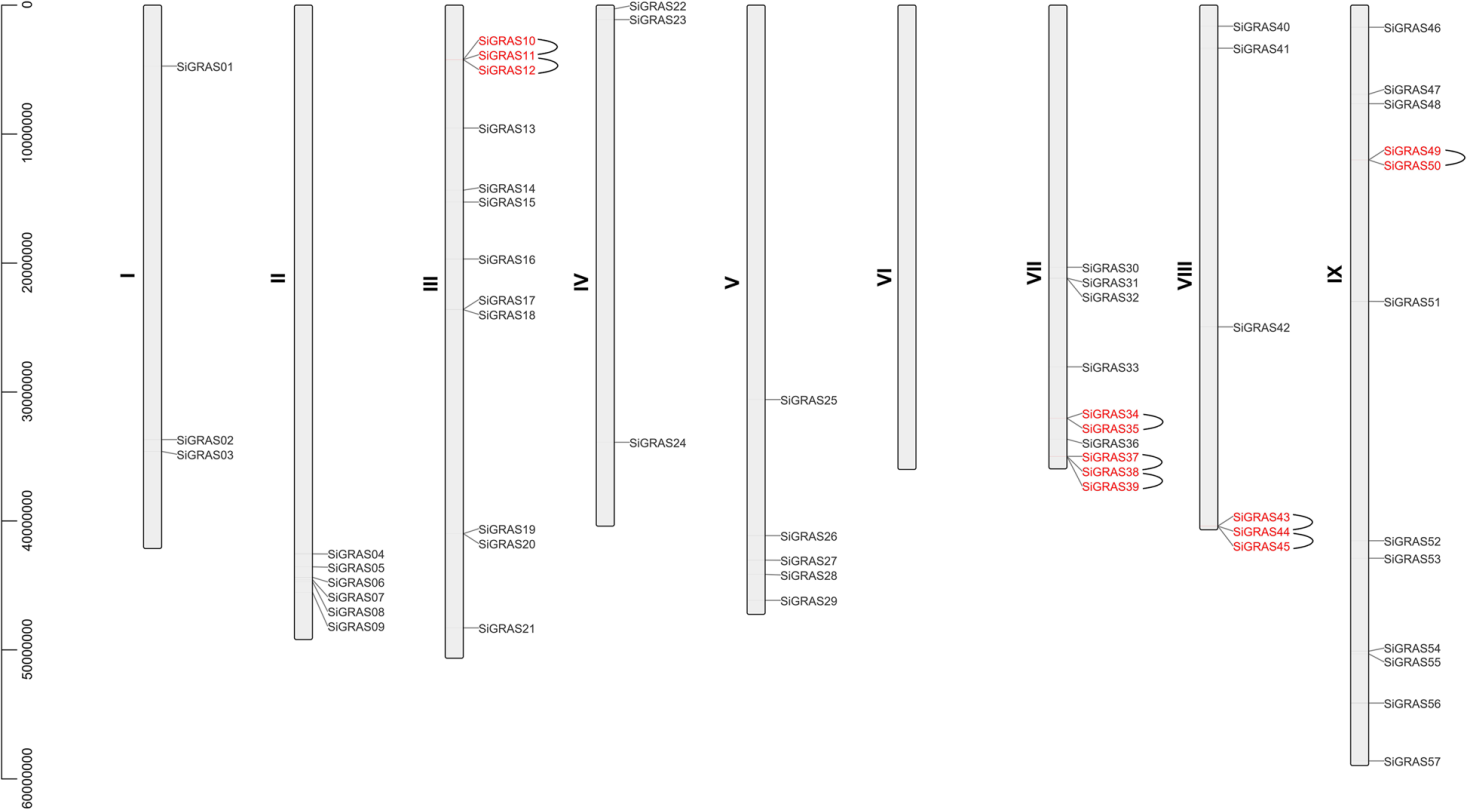
Schematic representations of the chromosomal distribution of the *S. italica GRAS* genes. Vertical bars represent the chromosomes of *S. italica*. The chromosome number is indicated to the left of each chromosome. The scale on the left represents chromosome length

The name of each SiGRAS gene name corresponds to its physical position from the top to the bottom of chromosomes 1 (Chr I) to 9 (Chr IX) of *S. italica* (Fig. 3, Tables S1 and S5). The distribution of the 57 *SiGRAS* genes on the chromosomes was uneven. Interestingly, *SiGRAS* genes were not found on Chr6 (Chr VI). Chr3 and Chr9 contained the largest number of *SiGRAS* genes (12 genes, ~ 21.05%), followed by Chr7 (10, ~ 17.54%); Chr1 and Chr4 contained the fewest SiGRAS genes (three each, ~ 5.26%). Chr5, Chr2, and Chr8 contained five (~ 8.77%), six (~ 10.53%), and six (~ 10.53%) *SiGRAS* genes, respectively. A chromosomal region within 200 kb range containing two or more genes was defined as a tandem duplication event [46]. On Chr3, 7, 8, and 9, we found eight tandem duplication events involving 13 *SiGRAS* genes (Fig. 3). *SiGRAS11, SiGRAS38* and *SiGRAS44* each had two tandem repeat events (*SiGRAS11* and *SiGRAS10*/*SiGRAS12*; *SiGRAS38* and *SiGRAS37*/*SiGRAS39*; *SiGRAS44* and *SiGRAS43*/*SiGRAS45*). All *SiGRAS* genes that formed tandem repeat events belonged to the same subfamily. For example, *SiGRAS49* and *SiGRAS50* were tandem repeat genes that were clustered together in the LISCL subfamily (Fig. 3, Table S1). Ten of the thirteen tandem repeat genes were derived from the LISCL subfamily, which suggested that it played a major role in *GRAS* genes expansion in evolution. Furthermore, the LISCL subfamily is also the subfamily with the highest number of members. Only *SiGRAS37*, *SiGRAS38*, and *SiGRAS39* were from the SCL3 subfamily.

**Fig. 4:**
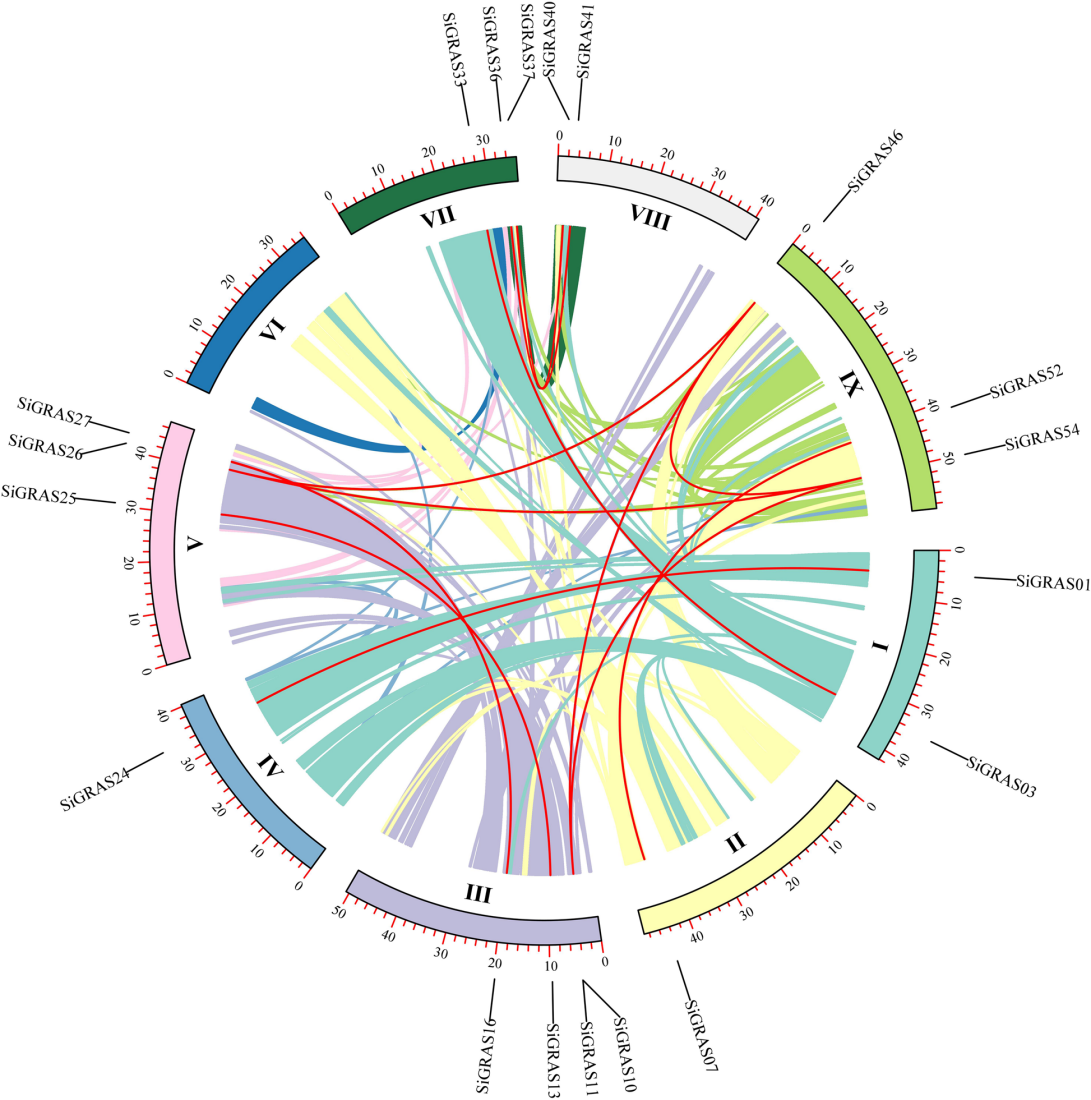
Schematic representations of the chromosomal distribution and segmental duplication relationships of *S. italica GRAS* genes. Colored lines indicate all synteny blocks in the *S. italica* genome and the red lines indicate duplicated *GRAS* gene pairs. The chromosome number is indicated at the bottom of each chromosome

**Fig. 5:**
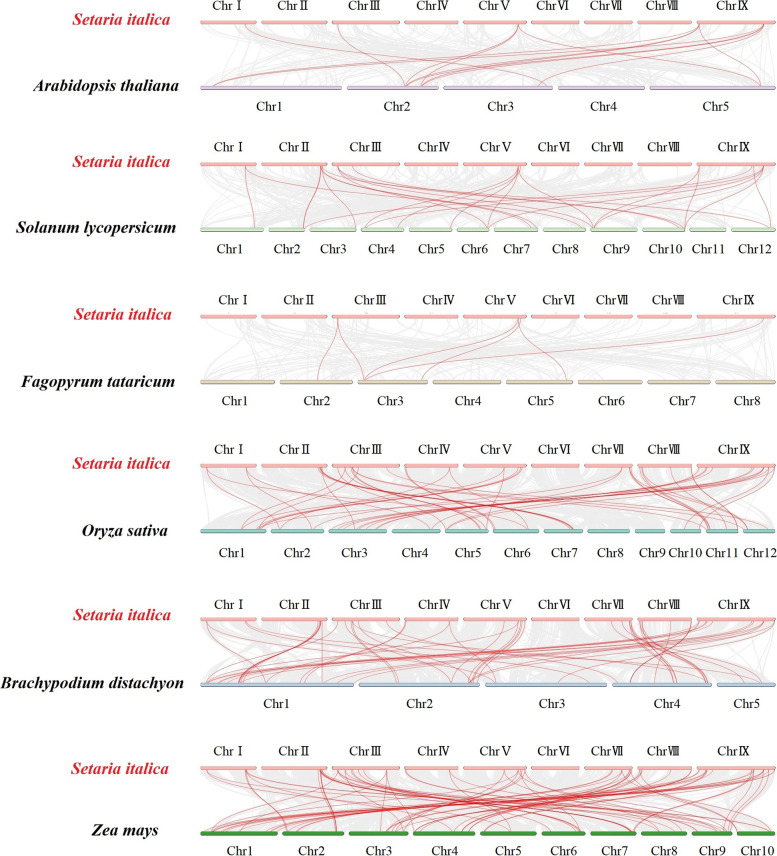
Synteny analyses of the *GRAS* genes between *S. italica* and six representative plant species. Gray lines on the background indicate the collinear blocks within *S. italica* and other plant genomes; red lines highlight the syntenic *S. italica GRAS* gene pairs

In addition, there were 12 pairs of segmental duplications in the *SiGRAS* genes (Fig. 4, Table S6). As shown in Fig. 5, 19 (~ 33.33%) paralogs were identified in the *SiGRAS* gene family, which indicated an evolutionary relationship for these *GRAS* genes. Although located on different chromosomes, *SiGRAS46*/*SiGRAS54* is closely related to *SiGRAS26*, which may be evidence of a gradual expansion of the LISCL subfamily. The segmental duplications were unevenly distributed in nine linkage groups (LG) of *S. italica*. LG3 had four *SiGRAS* genes, whereas LG2 and LG5 had only one *SiGRAS* gene. All of the segmental repeat gene pairs came from the same subfamily, (Table S6). For example, *SiGRAS46* and *SiGRAS54/SiGRAS26* were segmental paralogs clustered together in the LISCL subfamily, which had the largest number of linked genes (5/19, ~ 26.32%). In addition, the PAT1 subfamily had four segmental duplications, whereas the DELLA, HAM, LAS, SCL3, and SHR subfamilies had only one pair of segmental duplication.

### Synteny analysis of *SiGRAS* genes

To further infer the syntenic relationships of the *GRAS* genes, we constructed six comparative syntenic maps of *S. italica* with six representative species: three dicotyledons (*A. thaliana*, *S. lycopersicum* and *F. tataricum*) and three monocotyledons (*O. sativa*, *B. distachyon,* and *Z. mays*) (Fig. 5, Table S7). We found that a total of 47 *SiGRAS* genes showed collinear relationships with those in *Arabidopsis* (6), *F. tataricum* (4), tomato (15), *B. distachyon* (37), rice (38), and maize (59). The number of orthologous gene pairs between foxtail millet and the other six species (*Arabidopsis*, tomato, *F. tataricum, B. distachyon*, rice, and maize) was 13, 25, 6, 53, 52, and 81, respectively.

Some *SiGRAS* genes were found to exist in at least one pair of collinear genes in six plants, such as *SiGRAS10* with *AT2G29060*/*Solyc10g086530*/*FtPinG0201759800*/*BGIOSGA018951*/*BRADI_1g03620v3*/*Zm00001d028603*, which suggested that these orthologous genes already existed before the ancestral divergence. As expected, many orthologous gene pairs (with 12 *SiGRAS* genes) identified between *S. italica* and *B. distachyon*/rice/maize were not identified in *S. italica* and *Arabidopsis*/ *tomato*/*F. tataricum,* such as *SiGRAS13* with *BGIOSGA001121*/*BRADI_2g45117v3*/*Zm00001d044065*, and *SiGRAS25* with *BGIOSGA001121*/*BRADI_2g45117v3*/*Zm00001d044065.* This suggests that these orthologous pairs may have been gradually formed after the independent differentiation of monocotyledons (Table S7). To better observe the evolutionary constraints of the 57 *SiGRAS* genes, the *SiGRAS* genes were subjected to the Tajima D neutrality test [47, 48]. The calculated D of 6.77 deviated significantly from 0, suggesting that the *SiGRAS* gene family might have been involved in purification and selection pressure during the evolution process (Table S8).

### Evolutionary analysis of the SiGRAS and GRAS genes of several different species

To analyze the evolutionary relationship of the trihelix family of GRAS proteins among *S. italica* and six plants (*A. thaliana*, *F. tataricum*, *S. lycopersicum*, *B. distachyon*, *O. sativa*, and *Z. mays*), an unrooted neighbor-joining (NJ) tree was constructed. The cluster tree contained ten conserved motifs according to the MEME web server relative to the protein sequences of the 57 identified *SiGRAS* genes and the GRAS genes of the six other plants (Fig. S2, Table S3).

As shown in Fig. S2, the GRAS proteins of *S. italica* tended to cluster with those of *O. sativa* and *Z. mays*, which suggested that these GRAS proteins were more closely related. Furthermore, some homologous GRAS proteins had similar motifs. Most GRAS proteins of these seven plants contained motifs 7, 5, 2, 6, 9, 3, and 8. In addition, several motifs existed in specific topologies, such as motif 4. Interestingly, some motifs tended to be located in a specific composition. For example, motif 5 was always located between motifs 7 and 2, motif 10 was always located at the start of the pattern, and motif 8 was almost always located at the end of the pattern. In general, the GRAS proteins of *O. sativa*, *Z. mays*, and *S. italica* on the same branch had similar motif compositions. Similar compositions tended to cluster in specific GRAS protein subfamilies, which indicated potential functional similarities among those proteins.

### Expression patterns of SiGRAS genes in several organs

**Fig. 6:**
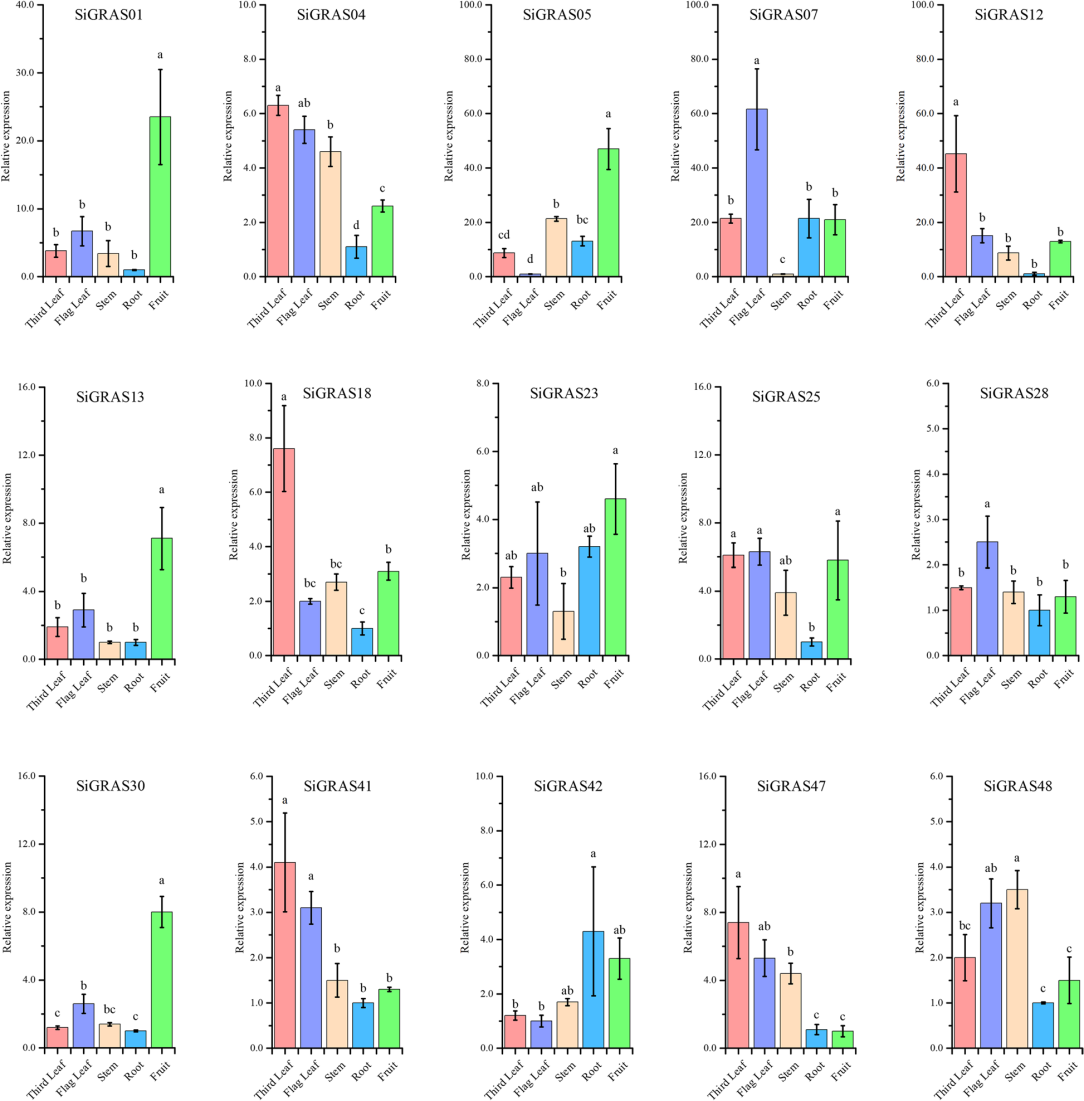
Expression patterns of 15 *S. italica GRAS* genes in the third leaf, flag leaf, root, stem, and fruit organs were examined by qRT-PCR. Detail: *SiGRAS01*, *SiGRAS04*, *SiGRAS05*, *SiGRAS07*, *SiGRAS12*, *SiGRAS13*, *SiGRAS18*, *SiGRAS23*, *SiGRAS25*, *SiGRAS28*, *SiGRAS30*, *SiGRAS41*, *SiGRAS42*, *SiGRAS47*, and *SiGRAS48* are from subfamily LAS, PAT1, SCR, SHR, LISCL, DELLA, SCL3, DLT, DELLA, OS4, OS19, HAM, OS43, SCL4/7, and DELLA, respectively. As far as possible, these subfamilies have distant clustering relationships and significant differences in their amino acid structures. Error bars were obtained from three measurements. Lowercase letters above the bars indicate significant differences (α = 0.05, LSD) among the treatments. The SE is selected as the value of the bar. The same is below

To investigate the potential roles of these *SiGRAS* genes, real-time polymerase chain reaction was used to detect the expression of 15 individual members from different subfamilies in five organs (third leaf, flag leaves, stems, roots, and fruits) (Fig. 6). The results showed that the expression patterns of *SiGRAS* genes varied greatly in different tissues and organs, suggesting that they have multiple functions in foxtail millet growth and development. Some genes showed preferential expression in the detected tissues. Four genes (*SiGRAS01*, *SiGRAS05*, *SiGRAS13*, *SiGRAS30*) were most highly expressed in the fruits, five genes (*SiGRAS04, SiGRAS12, SiGRAS18, SiGRAS41*, and *SiGRAS47*) were most highly expressed in the third leaves, and *SiGRAS07* and *SiGRAS28* were most highly expressed in the flag leaves. In addition, *SiGRAS48* and *SiGRAS42* showed high expression in the stems and roots, respectively.

Further, some *SiGRAS* genes may regulate the fruit development of foxtail millet, thus affecting its nutritional composition and development rate. Therefore, we explored to study the expression of these 15 Si*GRAS* genes at 18 (early filling stage), 25 (middle filling stage), and 32 (initial maturity stage) days post-anthesis (DPA) to identify the genes that may regulate *S. italica* fruit development (Fig. S3). The results showed that the expression levels of most *SiGRAS* genes were different in the fruits and glume during the three stages of fruit development. *SiGRAS18* expression increased with foxtail millet fruit development, whereas the expression level of *SiGRAS41* expression decreased with fruit development. Interestingly, the expression level of most genes (*SiGRAS01*, *SiGRAS05*, *SiGRAS07*, *SiGRAS13*, *SiGRAS23*, *SiGRAS25*, *SiGRAS30*, and *SiGRAS42*) were the highest at 25 DPA, while the expression level of *SiGRAS12* and *SiGRAS47* was at its lowest level at 25 DPA. In the glume, the expression level of six genes (*SiGRAS04*, *SiGRAS05*, *SiGRAS07*, *SiGRAS28*, *SiGRAS41*, and *SiGRAS42*) decreased with fruit development, whereas the expression of four genes (*SiGRAS01*, *SiGRAS18*, *SiGRAS30*, and *SiGRAS48*) increased.

The expression patterns of SiGRAS genes were coordinated in several plant organs, which indicates that their roles may be synergistic (Fig. S4). Most *SiGRAS* genes showed significant positive correlations; for example, five genes (*SiGRAS05*, *SiGRAS07*, *SiGRAS13*, *SiGRAS23,* and *SiGRAS25)* were significantly positively correlated. *SiGRAS04*, *SiGRAS28,* and *SiGRAS42* also showed a significantly positive correlation. However, many pairs of *SiGRAS* genes (*SiGRAS18* and *SiGRAS05/SiGRAS07/SiGRAS23*/*SiGRAS25*; *SiGRAS47* and *SiGRAS13*/*SiGRAS25*; *SiGRAS12* and *SiGRAS23*) were significantly negatively correlated.

### Effects on grain development and DELLA subfamily expression of after paclobutrazol treatment

**Fig. 7:**
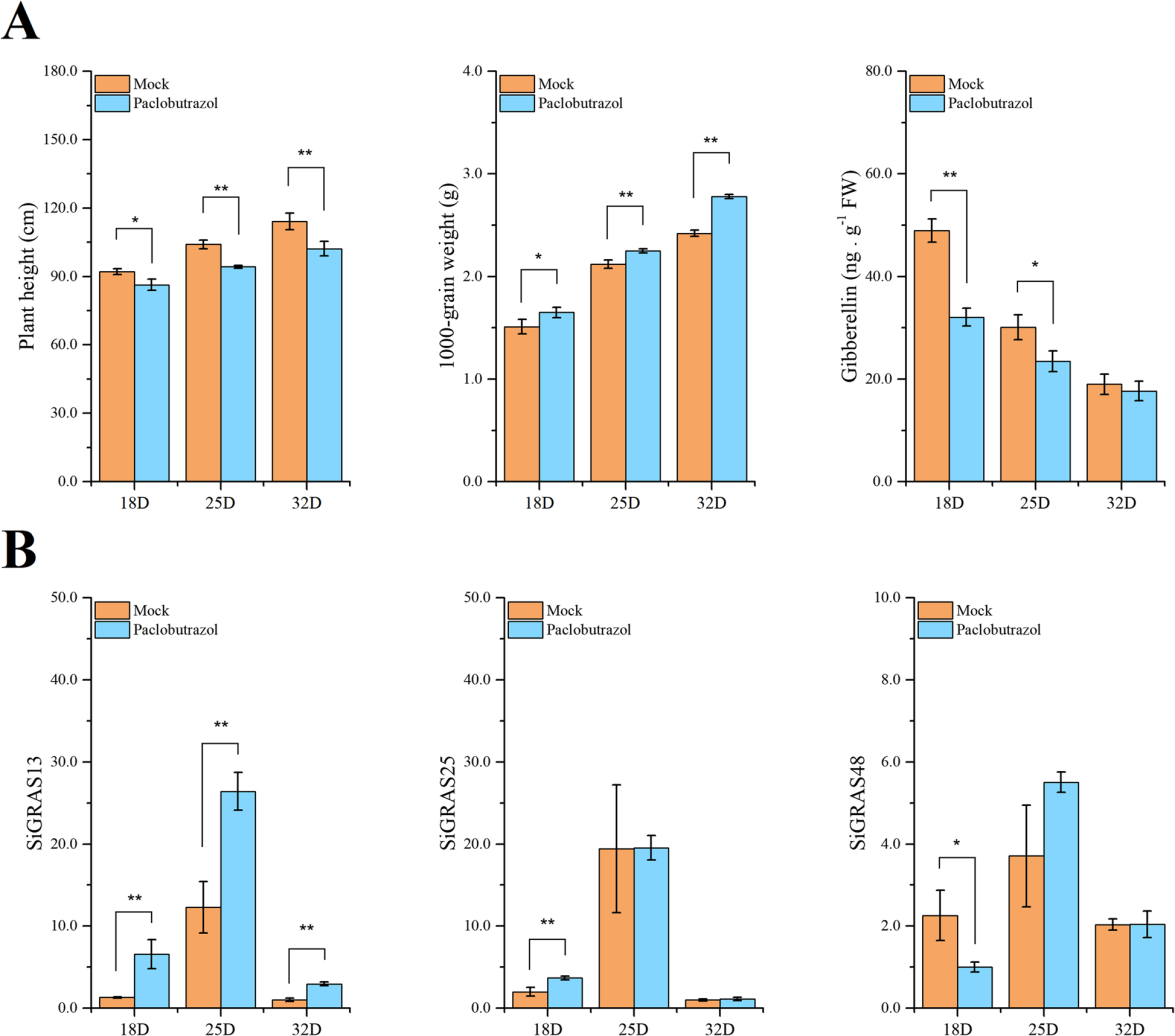
Fruit development of *S. italica* under exogenous paclobutrazol treatment. **A** The plant height, 1000 grain weight, and gibberellin content during fruit development. **B** Differences in the expression of DELLA subfamily genes under exogenous paclobutrazol treatment during fruit development. Mock: the same amount of water treatment, paclobutrazol: 250 mg·L^− 1^ paclobutrazol treatment. Error bars were obtained from three measurements. Small letter(s) above the bars indicate significant differences (α = 0.05/0.01, LSD) among the treatments. * and ** indicate significant correlations at the 0.05 and 0.01 levels, respectively

The plant height, 1000-grain weight, and gibberellin content were observed at different stages of grain development in foxtail millet after paclobutrazol (Fig. 7A). The results showed that the plant height of foxtail millet was decreased by paclobutrazol treatment, whereas the 1000-grain weight was increased significantly, particularly in the later stage of grain development. Additionally, the endogenous GA content of both the mock and paclobutrazol treatment groups decreased during grain development. We found that the GA content of the paclobutrazol treatment group dropped to a lower level more dramatically in the early filling stage (18 DPA), but there was no significant difference at the initial maturity stage (32 DPA).

We further investigated the expression of DELLA subfamily genes (*SiGRAS13*, *SiGRAS25*, and *SiGRAS48*) when foxtail millet was treated with 250 mg/L exogenous paclobutrazol (Fig. 7B) [42]. The experimental group was treated with paclobutrazol, while the control group was treated with the same amount of water. The results showed that the expression patterns of the three DELLA genes were significantly altered during fruit development. The expression of all genes showed an initial increasing trend followed by a decrease, and the expression level reached the maximum value at 25 DPA. *SiGRAS25* expression was increased at 18 DPA, compared with that in the control group, while *SiGRAS48* expression decreased at the early filling stage. Notably, the expression of *SiGRAS13* increased significantly during the whole grain development stage, and its response to external paclobutrazol was more significant.

### Expression patterns of *SiGRAS* genes in response to different abiotic stresses

To further determine whether the expression of *SiGRAS* genes is influenced by different abiotic stresses, 15 SiGRAS genes were examined for their expression under eight abiotic stresses: acid (HCl0.1 mol/L), alkali (0.2 mol/L), polyethylene glycol (PEG, 10%), NaCl (5%), heat (40 °C), cold (4 °C), flooding and darkness. Overall, many SiGRAS genes were significantly induced/repressed by the different forms of stress (Fig. S5). The expression levels of SiGRAS genes changed with time or in different organs, depending on the specific treatments. For example, under heat stress, the expression of *SiGRAS04*, *SiGRAS05*, *SiGRAS07*, *SiGRAS12*, *SiGRAS13*, *SiGRAS23*, *SiGRAS25,* and *SiGRAS47* were first significantly upregulated in the roots and stems but was then downregulated. Under cold stress, *SiGRAS18* expression was significantly downregulated in the stems and leaves at 24 h, whereas it was significantly up-regulated in the roots. Under NaCl stress, *SiGRAS41* expression was significantly upregulated in the roots, but downregulated in stems. Interestingly, several SiGRAS members showed opposing expression patterns under different treatments. The expression level of *SiGRAS41* was upregulated in stems and leaves in heat treatment, whereas its expression pattern was reversed by cold stress. The expression of some genes showed similar patterns under different stress treatments. For example, *SiGRAS04* expression was initially unchanged but was then significantly upregulated in the roots, stems, and leaves by the heat and cold treatments. Some other genes showed changes in specific organs. For instance, *SiGRAS23* responded significantly to heat and cold treatment in the leaves and roots, and *SiGRAS42* responded significantly to acid and alkali treatment in the roots (*P* < 0.05). In most treatments, the expression of *SiGRAS13* (DELLA) increased significantly. Furthermore, correlations between *SiGRAS* gene expression patterns were observed (Fig. S6). Most of the *SiGRAS* members were weakly related. However, a few *SiGRAS* genes were significantly positively correlated, such as *SiGRAS05* with *SiGRAS07*, and *SiGRAS28* with *SiGRAS48* (*P* < 0.05).

## Discussion

### *SiGRAS* gene structure and evolutionary analysis

The *GRAS* gene family of millet was systematically analyzed, and a total of 57 *SiGRAS* genes were identified. All the proteins showed significant structural differences, which indicated the high complexity of the *GRAS* family. The length of GRAS protein ranges from 248 to 912 amino acids, which account for the length and sequence variability of GRAS [6–9]. The ratio of *SiGRAS* genes to the total number of genes in the *S. italica* genome (~ 38,801 genes) [49, 50] was approximately 0.17%, which was more than that in *Arabidopsis* (0.11%) [15], rice (0.15%) [17], tomato (0.15%) [37], *C. sativus* (0.14%) [44] and Tartary buckwheat (0.14%) [42], but less than in *Carica papaya* (0.31%) [45] and *Medicago truncatula* (0.29%) [12]. The GRAS proteins of millet were divided into 13 subfamilies in the phylogenetic analysis, including LISCL, SCL4/7, DELLA, HAM, SHR, PAT1, SCR, OS4, SCL3, LAS, OS19, and DLT (Fig. 1). At least one SiGRAS protein has been identified in each *Arabidopsis* subpopulation, suggesting that these GRAS subfamilies have not been lost during long-term evolution and may play some fundamental biological functions [15, 17]. Our study supports that the hypothesis that the separation of the *GRAS* family may precede the separation of millet and *A. thaliana* [15]. As expected, some SiGRAS proteins (SiGRAS28, SiGRAS30, and SiGRAS42) are classified into rice specific subfamilies, which suggested that further differentiation of the GRAS family in monocotyledons may result in the formation of independent branches [15, 17]. As such, the unique physiological functions of these SiGRAS proteins need further study. Among the 13 subfamilies, the LISCL subfamily has the highest number of members (18, ~ 31.6%). Meanwhile, the DLT (*SIGRAS23*), SCL4/7 (*SIGRAS47*), OS19 (*SIGRAS30*), OS4 (*SIGRAS28*) and OS43 (*SIGRAS42*) subfamilies had the fewest members (only one *SiGRAS*). Further expansion of the LISCL subfamily is supported by high homology among members (*SiGRAS26*, *SiGRAS46*, and *SiGRAS54*) of several different chromosomes. Similar to the GRAS subfamilies of other plants, such as *A. thaliana* [15], rice [17], buckwheat [42], and sorghum [43], the different subfamilies may have different differentiation abilities in the long-term evolutionary process. Evolutionary differences in these genes may be important for their various functions in species. However, there is insufficient research to show that the process of environmental adaptation is related to differences in differentiation among subfamilies.

The highly variable N-terminus of GRAS protein constitutes IDRs, which contain molecular recognition features and some easily exchanged gene fragments. These functional regions can complete the recognition of their specific binding objects through disordered and ordered transformation [1, 51]. SiGRAS proteins showed abundant N-terminal differences, which indicated their functional diversity of SiGRAS proteins (Fig. S1). The VHIID region of all subfamilies except the LISCL subfamily is relatively conserved, especially in the histidine “H” and aspartic acid “D” of amino acid residues. Meanwhile, the relative variability of valine “V” and isoleucine “I” was speculated to be caused by gene mutations [52, 53]. Nevertheless, the same subfamilies have similar amino acid structures, and they may perform similar physiological functions [42]. In addition, some GRAS proteins (SiGRAS31 SiGRAS32, SiGRAS43, SiGRAS45), which were attributed to the LISCL subfamily, did not have conserved the histidine “H” and aspartic acid “D” in the VHIID region. This discrepancy may be evidence of further differentiation of the *GRAS* genes, with new structures giving rise to new functions. A similar phenomenon has been found in sorghum [43], which is different from that in *Arabidopsis* [15]. We speculate that the high activity of the LISCL subfamily leads to structural differentiation, which may be due to the instability of amino acids. This phenomenon may be the reason for the expansion of the subfamily, leading it to become the largest subfamily. Further, the SiPAT1 and SiSCR subfamilies have the most conserved structures, which are similar in the lower mosses and ferns and the higher species [54]. We also observed some domain loss events in *SiGRAS19* and *SiGRAS20* (Fig. S1), both of which were classified into the LISCL subfamily, which may be the result of heterotopic or inversion of chromosome fragments [55]. Domain gain and loss is a driving force for gene family expansion, which often occurs in monocotyledonous plants such as rice and maize [56, 57]. The proportion of SiGRAS genes without introns (37, ~ 64.9%) was higher than that of rice (55%) [15] and poplar (54.7%) [32], lower than that of Tartary buckwheat (41, ~ 87%) [42] and close to that of sorghum (66.67%) [43]. Genes without introns are also found in other large gene families, such as the F-box transcription factor gene family [58] and DEAD-box RNA helicase [59]. *GRAS* genes in plants may originate from prokaryotic genes through horizontal gene transfer and repeated events during evolution; as such, a large number of *GRAS* gene family members are intron-free genes [41]. From the perspective of evolution, the existence of introns can increase the length of genes and the frequency of intergene recombination, thereby conferring a positive effect on evolution [60]. However, intron-free genes are not isolated during transcription and translation, can continuously encode proteins, and tend to respond quickly to environmental changes [61, 62].

The tandem repetitions and fragment replication events play key roles in the expansion of *GRAS* gene family in foxtail millet. The proportion of GRAS protein in foxtail millet is higher than that in *A. thaliana* and rice [15](~ 0.17%), which indicates that there may be more gene repetition events or higher retention frequency after gene replication in millet. We found eight tandem repeat events in the *SiGRAS* gene family involving 13 members (~ 22.8%), which is higher than that in *Arabidopsis* (2/34) [15], plum (10/45) [63], and tomato (15/53) [37], but lower than that in poplar (40/106) [32] and sorghum (25/81) [43]. Notably, most tandem duplicates of *SiGRAS* genes were from the same subfamily, and mainly occurred in the LISCL subfamily (10, ~ 76.9%). This indicates that members of some GRAS protein subfamilies have a higher degree of preference for replication events, and these genes do not show great structural differences after replication events [43]. In addition, this study found that fragment duplication (29 *SiGRAS* genes, ~ 33.3%) contributed slightly more to the increase of *GRAS* members in millet than tandem duplication, which was similar to that in tartary buckwheat [42] and sorghum [43]. This may indicate that the LISCL subfamily may have a stronger expansion in plant evolution, not just in the evolution of C4 plants. All SiGRAS genes differ in genetic structure, while members of the same subfamily have similar genetic structures. This further supports that *SiGRAS* genes in the common taxa share a common evolutionary origin and molecular function, making this an effective and practical method for predicting unknown protein functions [42].

### Expression patterns and functional prediction of the *SiGRAS* genes

*SiGRAS07* showed the highest expression in the flag leaves (Fig. 6), which was consistent with the expression pattern of the homologous gene *AT4G37650*. *AT4G37650* (*AtSHR*) may play a key role during the visible and flowering stages of leaves and the mature plant embryo stage in *A. thaliana* [64]. The transcription levels of *SiGRAS04* and the homologous gene *AT5G48150* were both high in the stems and leaves, and the *AT5G48150* is required for maintenance of shoot apical meristem and the youngest primordia in *A thaliana* [65]. *SiGRAS13*, a member of the DELLA subfamily, also demonstrated higher expression during the fruit-filling stage, suggesting an important role in the development of fruits of foxtail millet. *SiGRAS05* of the SCR subfamily was highly expressed in fruit, and the SHR-SCR-SCL23 module plays a key role in the formation of endodermis in *A. thaliana* [66]. However, specific functions need to be analyzed through in-depth experiments. Simultaneously, we also found that most *SiGRAS* members genes were expressed at higher levels in the middle filling stage (25 DPA). This finding is different from that shown by dicotyledons buckwheat [42] and castor beans [67], which indicates that these genes may be involved in middle fruit development.

The Gibberellin could be detected in the whole fruit development stage of foxtail millet (Fig. 7), which gradually decreased from 18DPA (48.92 ng·g^− 1^) to 32DPA (18.98 ng·g^− 1^). Therefore, we hypothesized that gibberellin is produced in young fruits of foxtail millet immediately after fertilization to promote the fruit initiation process [68]. Comparing the members of the DELLA subfamily (*SiGRAS13*, *SiGRAS25*, and *SiGRAS48*), the expression of SiDELLAs during middle fruit development was significantly higher than that at the early (18 DPA) and late (32 DPA) stages. Therefore, we hypothesized that DELLA genes may play a role during the middle development stages of fruits. Paclobutrazol, a triazole plant growth regulator, regulates plant tissue and fruit development by inhibiting GA biosynthesis through the regulation of DELLA transcription [43]. The plant height was significantly reduced at the grain filling stage in the paclobutrazol treatment group compared to that in the mock group. Similarly, in wheat, plant height and stem length were decreased after treatment with paclobutrazol. At the same time, the stem diameter, stem plumpness, and basal internode wall thickness were significantly increased in the paclobutrazol treatment group, leading to higher stem strength and higher lodging resistance index (CLRI) [69]. Then, the expression levels of the DELLA subfamily (*SiGRAS13*, *SiGRAS25*, and *SiGRAS48*) in the paclobutrazol treatment group were further analysed in *S. italica* (Fig. 7B). The expression patterns of all DELLA genes were significantly changed in the paclobutrazol treatment group compared to that in the mock group, especially in the early filling stage. The expression levels of *SiGRAS25* and *SiGRAS48* changed significantly at 18 DPA, which indicated that they may be sensitive in the early stage. After paclobutrazol treatment, the expression level of *SiGRAS13* expression was significantly increased throughout fruit development. Meanwhile, the sensitivity of *SiGRAS13* to paclobutrazol treatment was higher than that of *SiGRAS25* and *SiGRAS48*. Therefore, we speculate that *SiGRAS13* may has potential value in the breeding of *S. italica*. We found that paclobutrazol had significant inhibitory effects on gibberellin synthesis, especially at the early filling stage (18DPA). We hypothesized that the expression patterns of DELLA members may be influenced by the down-regulation of gibberellin (Fig. 7). At the same time, different DELLA genes may have different responses to gibberellin, most of which change significantly at the early filling stage, whereas some members have long-term responses (*SiGRAS13*). We hypothesized that the decrease of GA content relieved the inhibition of DELLA protein expression, and the plant height of foxtail millet was significantly inhibited. However, the application of paclobutrazol increased the 1000-grain weight of foxtail millet, which may be due to the increased accumulation of photoassimilate products [70].

As a drought-tolerant crop, foxtail millet may regulate its adaptation to the environment through complex endogenous networks and transcriptional signals, and similar conclusions have also been reached for poplar [32], sorghum [43], and Tartary buckwheat [42]. Previous studies have found that some members of the same subfamily with the same motif may have similar physiological functions [43]. Therefore, we can further speculate the function of SiGRAS genes, which needs to be further verified in experiments. *AT5G41920* and *SiGRAS05* both belonged to the SCR subfamily, had similar motif compositions, and were expressed preferentially under dehydration stress in *A. thaliana* [71]. Similarly, the expression of *SiGRAS05* was significantly upregulated in the roots under PEG stress, which may enhance the adaptability of foxtail millet to the environment in a similar pattern. *SiGRAS04* was classified under the PAT1 subfamily, and its expression was increased in the roots, stems, and leaves when under stress from NaCl, heat, cold, and flooding. *VaPAT1*, a GRAS gene from *Vitis amurensis*, acts as a stress-induced GRAS gene and enhances cold, drought, and salt tolerance of transgenic *Arabidopsis* by regulating the expression of a series of stress-related genes [72]. Interestingly, *AT1G50600*, *AT2G04890*, *AT4G17230,* and *AT5G48150*, which belong to the PAT1 subfamily, are all involved in the optical signaling pathway and are highly expressed in the leaves [26]. Furthermore, *SiGRAS04* had the same motifs and gene structures as these genes. Under dark conditions, *SiGRAS04* expression was rapidly and significantly upregulated in the leaves and stems, which may help regulate photosynthesis and respiratory balance; however, this needs to be further verified by future experiments. *SiGRAS47* responded significantly to different abiotic stresses in roots, and its expression was increased in 24 h during the seven stresses (acid, NaCl, heat, cold, flooding, and darkness). *PeSCL7*, a member of the SCL subfamily in poplars, enhances drought and salt resistance at the *Arabidopsis* seedling stage by promoting root development and reducing water loss rate [32]. *SiGRAS13*, *SiGRAS25*, and *SiGRAS48* belonged to the DELLA subfamily and were involved in almost all of the abiotic stress responses, especially *SiGRAS13* and *SiGRAS25*. Thus, we can assume that DELLA protein mediated gibberellin signalling plays an important role in a variety of abiotic stress responses in foxtail millet, and different DELLA members may play regulatory roles to different degrees. In *A. thaliana*, the high expression level of DELLA expression can improve the activity of reactive oxygen species (ROS), protect cells from ROS damage under abiotic stress, and enhance the survival ability of plants [73]. There were significant differences in gene expression patterns under different subfamilies, nevertheless, the correlated heat maps suggested that some members may still be co-expressed (Fig. S6), such as *SiGRAS05* with *SiGRAS07*. In *Arabidopsis*, *SHR* may interact with *SCR* to form the SHR-SCR heterodimer through conserved GRAS domains [74], which are regarded as central regulators in the radial patterning in the roots [75]. These results suggested that *GRAS* gene family may regulate the tissue development process and abiotic stress response in foxtail millet, which needs further experimental verification.

## Conclusion

We first identified and analyzed the genome-wide *SiGRAS* gene family in *S. italica*. 57 *SiGRAS* genes were distributed on eight chromosomes and divided into 13 subfamilies. Furthermore, we found that segment duplications and tandem duplications contributed to the expansion of the *SiGRAS* gene family, and segment duplication may have a more important contribution. Multiple sequence alignment and gene structure analysis showed that most of the *SiGRAS* genes lacked introns, which indicated that *SiGRAS* genes were conserved to some extent. In addition, we analysed the expression of 15 *SiGRAS* genes in different tissues (root, stem, leaf, and fruit) and fruit development stages and under eight different forms of abiotic stress. The relationship among DELLA genes, gibberellin content and fruit development in foxtail millet were further investigated. Paclobutrazol treatment significantly down-regulated plant height and gibberellin content, but increased grain weight during whole-grain development. In addition, the expression level of *SiGRAS13* expression was upregulated after paclobutrazol treatment in *S. bicolor*; and *SiGRAS25* was sensitive to the eight abiotic stresses. These findings may be valuable considerations when breeding *S. italica*.

## Methods

### Gene identification

The entire Foxtail millet genome was downloaded from the Ensembl Genomes website (http://ensemblgenomes.org/). Foxtail millet GRAS sequences were obtained through two BLASTP methods [76, 77]. Firstly, the candidate GRAS proteins of foxtail millet were authenticated by a BLASTp search. Second, we downloaded the hidden Markov model (HMM) file corresponding to the GRAS domain (*PF03514*) from the Pfam protein family database (http://pfam.sanger.ac.uk/). The GRAS protein sequences were retrieved from the foxtail millet genomic database using HMMER3.0 with a cutoff of 0.01 (http://plants.ensembl. org/hmmer/index.html) [78]. The existence of the GRAS core sequences was confirmed by the PFAM and SMART programs (http://smart.embl-heidelberg.de/) [79, 80]. Finally, 57 SiGRAS genes were identified in the foxtail millet genome. Then, 57 SiGRAS proteins were used as initial queries in the NCBI protein database (https://blast.ncbi.nlm.nih.gov/Blast.cgi? PROGRAM = blastp&PAGE_TYPE = BlastSearch&LINK_LOC = blasthome) using BLASTp to verify the GRAS proteins. In addition, ExPasy (http://web.expasy.org/protparam/) was used to identify the basic features of the trihelix proteins of the GRAS genes of *S. italica* were identified: the sequence length, isoelectric point (pi), molecular weight (mws), and subcellular localization.

### GRAS structure

Based on the default parameters of ClustalW, the domain sequences of the characterized GRAS proteins of *A. thaliana* and rice were used to create multiple protein sequence alignments with the SiGRAS domain sequences of different subfamilies [81]. Then GeneDoc software and Mega7.0 were used to manually adjust the amino acid sequences of the GRAS domain in different subfamilies. Then, the conserved motif and structural differences of 57 SiGRAS proteins were analyzed [55, 82]. The exon-intron organization of GRAS genes of foxtail millet were determined by comparing predicted coding sequences with their corresponding full-length sequences using the online program Gene Structure Display Server (GSDS: http://gsds.cbi.pku.edu.cn) [83]. The conserved motifs in the identified SiGRAS proteins were identified using the MEME online program (http://meme.nbcr.net/meme/intro.html) [82]. The optimized parameters of motif width were employed as the following:the maximum number of motifs was 10, and the optimum width was 6 to 200 residues [43]. In addition, the software PlantCARE was used to predict the cis-acting elements of 57 *SiGRAS* genes in the upstream 2000 bp range (http://bioinformatics.psb.ugent.be/webtools/plantcare/html/?tdsourcetag=s_pcqq_aiomsg).

### Chromosomal distribution and gene duplication

All *SiGRAS* genes were mapped to *S. italica* chromosomes based on physical location information. The Circos program was used to process the chromosomal location information of the *SiGRAS* genes [82]. Multiple Collinearity Scanning toolkit (MCScanX) was adopted to analyze the *SiGRAS* gene duplication events, with the default parameters [84]. The homology of the *GRAS* genes between *S. italica* and the other six plants (*A. thaliana, F. tataricum, S. lycopersicum, B. distachyon, O. sativa* subsp*. indica,* and *Z. mays*) were analyzed using Dual Synteny Plotter (https://github.com/CJ-Chen/TBtools). The Tajima’s D Neutrality Test program of Mega7.0 software was used to further analyze the evolutionary constraints acting on *SiGRAS* genes [43].

### Phylogenetic analysis and classification of *SiGRAS* gene family

According to the classification of GRAS proteins of *Arabidopsis*, 57 GRAS proteins of *S. italica* were divided into several groups. In MEGA 7.0, the NJ tree was constructed used the Jukes-Cantor model. The phylogenetic tree was constructed with a bootstrap value of 1000 and assigned with Geneious R11 with BLOSUM62 cost matrix. In addition, the full-length amino acid sequences of the characteristic GRAS proteins derived from *A. thaliana, F. tataricum, S. lycopersicum, B. distachyon, O. sativa* subsp*. indica,* and *Z. mays* (Table S1) combined with the newly identified SiGRAS were used for phylogenetic analysis (UniProt: https://www.uniprot.org/).

### Plant materials, growth conditions, paclobutrazol treatment, and abiotic stress in *S. italica*

The foxtail millet accessions (Yugu 1) was widely cultivated in northern China and obtained from Prof. Cheng Jianping of Guizhou University. In 2020, ‘Yugu 1’ was planted in the greenhouse of the experimental base located at the farm at Guizhou University. Foxtail millet plants were grown in pots filled with soil and vermiculite (1:1) in a growth room with a 16 h/25 °C day and 8 h/20 °C night regime, with a relative humidity of 75%. We collected the five plants with good growth and similar growth conditions, respectively. The samples included the flag leaves, third leaves, roots, stems, fruits in the discoloration stage, and fruits and glumes in the three developmental stages (18 DPA, green fruit stage; 25 DPA, discoloration stage; and 32 DPA, initial maturity stage). In addition, the expression patterns of 15 SiGRAS genes under different stresses were further analysed. Foxtail millet plants at the seedling stage (28 days) were selected for the abiotic stress treatments, which included acid (HCL 0.1 mol/L), alkali (NaOH 0.2 mol/L), salt (5% NaCl), flooding (whole plant), drought (30% PEG6000), darkness (complete shading), heat (40 °C) and cold (4 °C). Five replicates were collected from each stress treatment, and the expression levels were analyzed at 0 h, 2 h, and 24 h, respectively. In addition, ‘Yugu 1’ materials with similar growth statuses were selected and sprayed with 50 mL paclobutrazol (250 mg·L^− 1^) during the germination period. The controls (mock) were sprayed with the same amount of water. The 1000-grain weight, plant height, gibberellin content, and gene expression level of DELLA subfamily of foxtail millet were further analyzed among control and paclobutrazol treatment at 18, 25, and 32 DPA after pollination. The samples were collected quickly put into liquid nitrogen, and stored at − 80 °C for subsequent analysis. Each sampling and stress treatment had five biological replicates. The samples were used for quantitative PCR (qPCR) with at least three technical repeats.

### Total RNA extraction, cDNA reverse transcription, and qRT-PCR analysis

The cDNA was produced with a 1 mg RNA sample using a PrimeScript RT Reagent Kit with gDNA Eraser (TaKaRa) and SYBR Premix Ex Taq II (TaKaRa) [85]. Total RNA was extracted using the RNA out Kit (TaKaRa) and treated with RNase-free DNase I to remove trace amounts of DNA. Gene-expression analysis of the selected genes was performed by qPCR, and the primers were designed using Primer 5.0 software (Table S9). We used the *actin* gene (*Si001873m.g*) as an internal control, which was stably expressed at each growth stage in almost all tissues. The experimental data were calculated according to the 2^−(ΔΔCT)^ method [86].

### Endogenous GA analysis

With reference to the method of Fan et al. [43], the GA content in foxtail millet fruits was determined. A fresh tissue sample of about 1 g fruit was collected and ground in liquid nitrogen. Fifty millilitres of 80% ethanol were added to the ground powder, which was then used for ultrasonic extraction three times for 1 h each time. The supernatant was concentrated once at a low temperature, and after mixing with water, an equal volume of n-butanol was added for extraction for 1 h. Finally, the n-butanol layer was dried under a stream of nitrogen (N_2_). Ten milligrams of the dried sample were accurately weighed and dissolved in 5 mL methanol. The dissolved solution was filtered using a 0.22 μm microporous membrane, and LC/MS was conducted for content detection.

### Statistical analysis

We processed and analyzed all the above data with variance analysis with JMP6.0 software (SAS Institute), and the means were compared by the least significant difference test (LSD) at significance levels of 0.05 and 0.01. The histogram was drawn using the Origin 2016 (OriginLab Corporation, Northampton, Massachusetts, USA). The correlation coefficients of the SiGRAS genes were defined using Sigmaplot 12.0 software based on the Pearson correlation program. The correlation coefficient was defined as significant at *P* < 0.05.

## Supplementary Information

**Additional file 1 **: **Table S1.** List of the 57 *S. italica GRAS* genes identified in this study.

**Additional file 2 **: **Table S2.** Subfamilies and protein sequences of *Arabidopsis* and rice.

**Additional file 3 **: **Table S3.** Analysis and distribution of the conserved motifs of GRAS proteins.

**Additional file 4 **: **Table S4.** Cis-regulatory elements in the promoter region of *GRAS* genes.

**Additional file 5 **: **Table S5.** Tandem duplication events of *S. italica GRAS* genes.

**Additional file 6 **: **Table S6.** The 12 pairs of segmental duplicates in *S. italica GRAS* genes.

**Additional file 7 **: **Table S7.** One-to-one orthologous relationships between *S. italica* and other plants.

**Additional file 8 **: **Table S8.** Results of Tajima's D neutrality test.

**Additional file 9 **: **Table S9.** Primer sequences for qRT-PCR.

**Additional file 10 **: **Figure S1.** Multiple sequence alignment of the GRAS domains of the members of 13 phylogenetic subfamilies of the *S. italica* GRAS protein family. The scheme at the top depicts the locations and boundaries of the LHR I, VHIID, LHR II, PFYRE, and SAW regions within the GRAS domain.

**Additional file 11 **: **Figure S2.** Phylogenetic relationships and motif compositions of the *S. italica* GRAS proteins with six different plant species (*A. thaliana, F. tataricum, S. lycopersicum, B. distachyon, O. sativa* subsp*. indica,* and *Z. mays*). Outer panel: Unrooted phylogenetic tree constructed using Geneious R11 with the NJ method. Innermost panel: Distribution of the conserved motifs in GRAS proteins. The differently colored boxes represent different motifs and their positions in each GRAS protein sequence. The sequence information for each motif is provided in Additional file 3: Table S3.

**Additional file 12 **: **Figure S3.** Expression patterns of 15 *S. italica GRAS* genes were examined during different fruit development stages: 18 DPA (early filling stage), 25 DPA (middle filling stage), and 32 DPA (initial maturity stage).

**Additional file 13 **: **Figure S4.** The correlations 15 *S. italica GRAS* genes in several plant organs. Positive number: positively correlated; negative number: negatively correlated. The correlation coefficients of *SiGRAS* genes were defined by software Sigmaplot 12.0 based on Pearson correlation program. The correlation coefficient is defined as significant correlation with *P*-value lower than 0.05.

**Additional file 14 **: **Figure S5.** Expression of 15 *S. italica GRAS* genes under different abiotic stresses (acid, alkali, PEG, NaCl, heat, cold, flooding and dark treatments) at the seedling stage. Error bars were obtained from three measurements. Lowercase letters above the bars indicate significant differences (α = 0.05, LSD) among the treatments.

**Additional file 15 **: **Figure S6.** The correlations 15*S. italica GRAS* genes in several abiotic stresses.

## Data Availability

The entire *Setaria italica* genome sequence information was from the Ensembl Genomes website (http://ensemblgenomes.org/). The *Setaria italica* materials (Yugu 1) used in the experiment were supplied by Prof. Cheng Jianping of Guizhou University. The datasets supporting the conclusions of this article are included in the article and its Additional files.

## Abbreviations

At: *Arabidopsis thaliana*
Os: *Oryza sativa*
AtGRAS: *Arabidopsis thaliana* GRAS
OsGRAS: *Oryza sativa* GRAS
*SiGRAS*: *Setaria italica* GRAS
qRT-PCR: Quantitative real-time polymerase chain reaction
TF: Transcription factor
CDS: Coding sequence
HMM: Hidden Markov Model
pI: Isoelectric point
BLAST: Basic local alignment search tool
LHR I: Leucine-heptad repeat I
LHR II: Leucine-heptad repeat II
SCR: SCARECROW
