# Supplementary figures and images for: Genome-wide investigation of the GRAS transcription factor family in foxtail millet (Setaria italica L.)
Source: BMC Plant Biol. 2021 Nov 3;21:508. doi: 10.1186/s12870-021-03277-y (PMC8565077; doi:10.1186/s12870-021-03277-y)

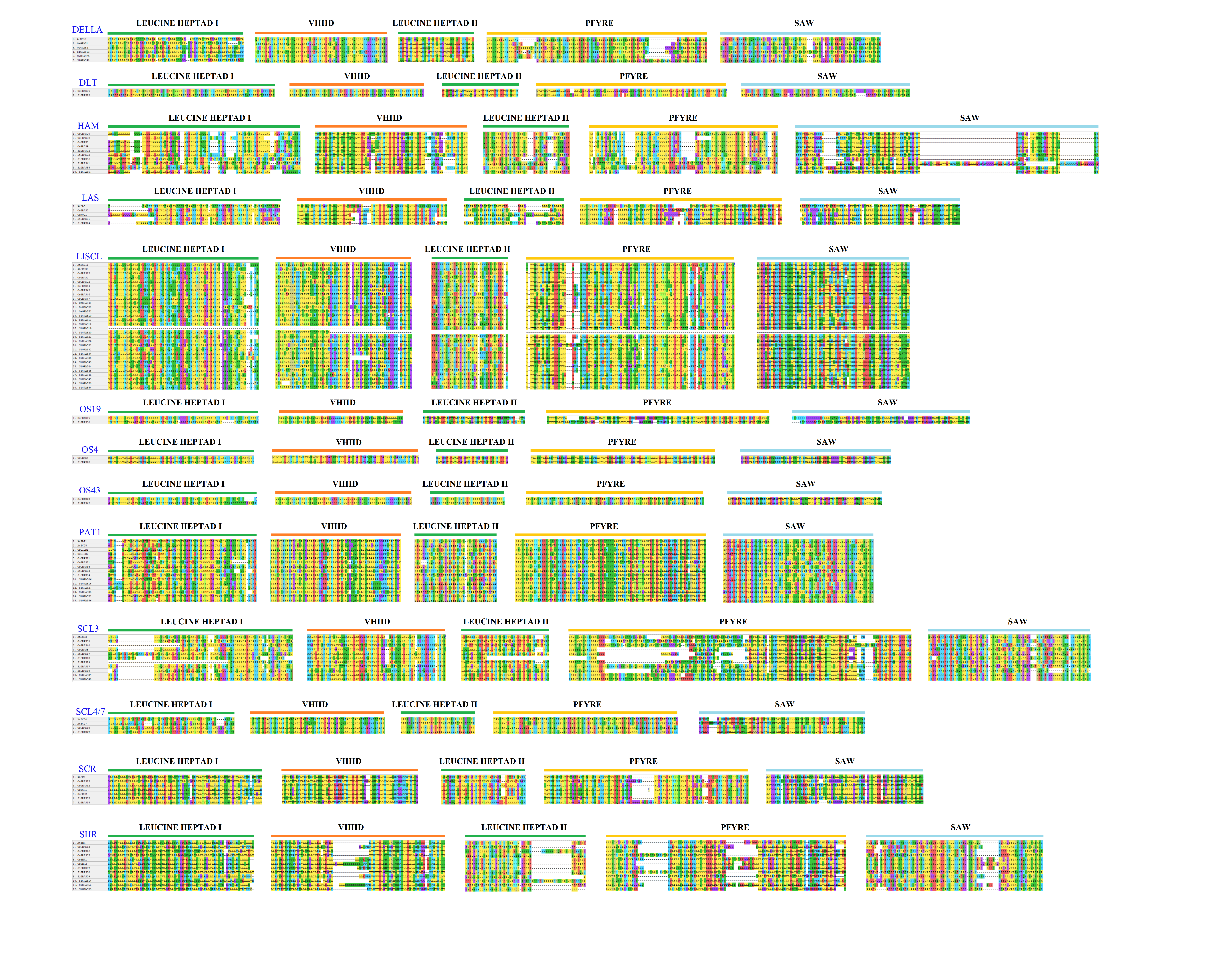

Supplement: Supplementary file 10 — Additional file 10 : Figure S1. Multiple sequence alignment of the GRAS domains of the members of 13 phylogenetic subfamilies of the S. italica GRAS protein family. The scheme at the top depicts the locations and boundaries of the LHR I, VHIID, LHR II, PFYRE, and SAW regions within the GRAS domain. [file 12870_2021_3277_MOESM10_ESM.png]

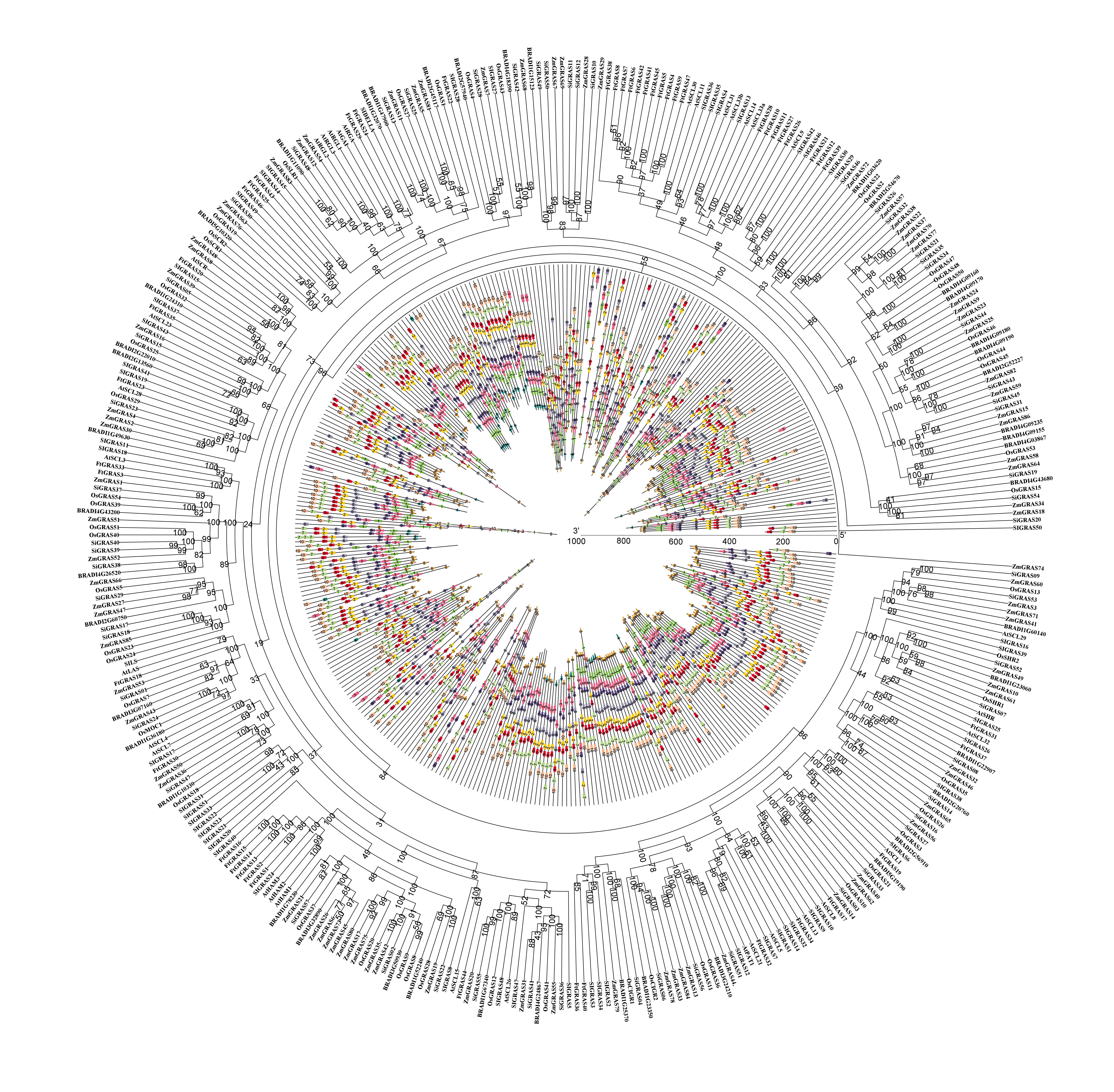

Supplement: Supplementary file 11 — Additional file 11 : Figure S2. Phylogenetic relationships and motif compositions of the S. italica GRAS proteins with six different plant species (A. thaliana, F. tataricum, S. lycopersicum, B. distachyon, O. sativa subsp. indica, and Z. mays). Outer panel: Unrooted phylogenetic tree constructed using Geneious R11 with the NJ method. Innermost panel: Distribution of the conserved motifs in GRAS proteins. The differently colored boxes represent different motifs and their positions in each GRAS protein sequence. The sequence information for each motif is provided in Additional file 3: Table S3. [file 12870_2021_3277_MOESM11_ESM.jpg]

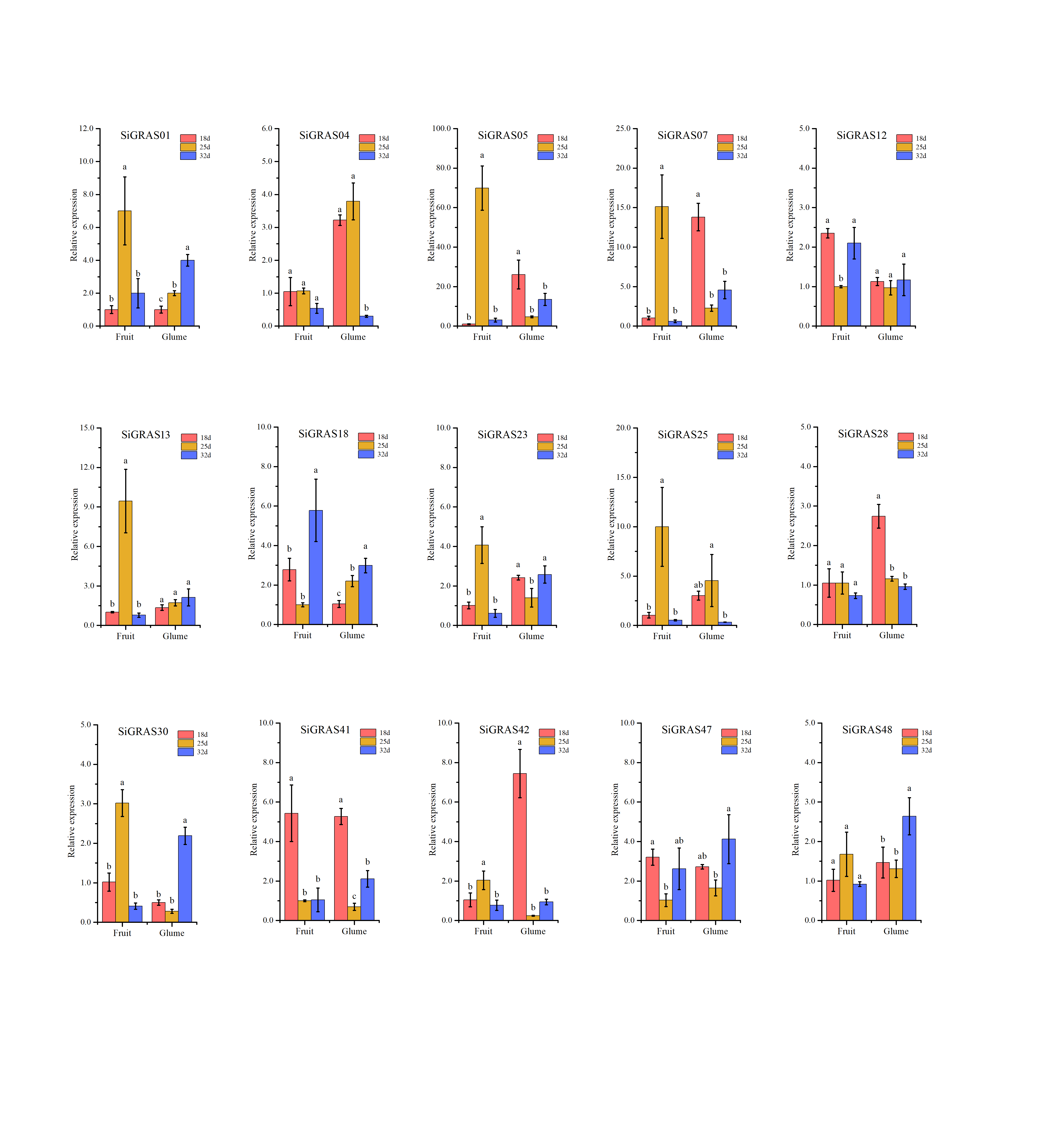

Supplement: Supplementary file 12 — Additional file 12 : Figure S3. Expression patterns of 15 S. italica GRAS genes were examined during different fruit development stages: 18 DPA (early filling stage), 25 DPA (middle filling stage), and 32 DPA (initial maturity stage). [file 12870_2021_3277_MOESM12_ESM.png]

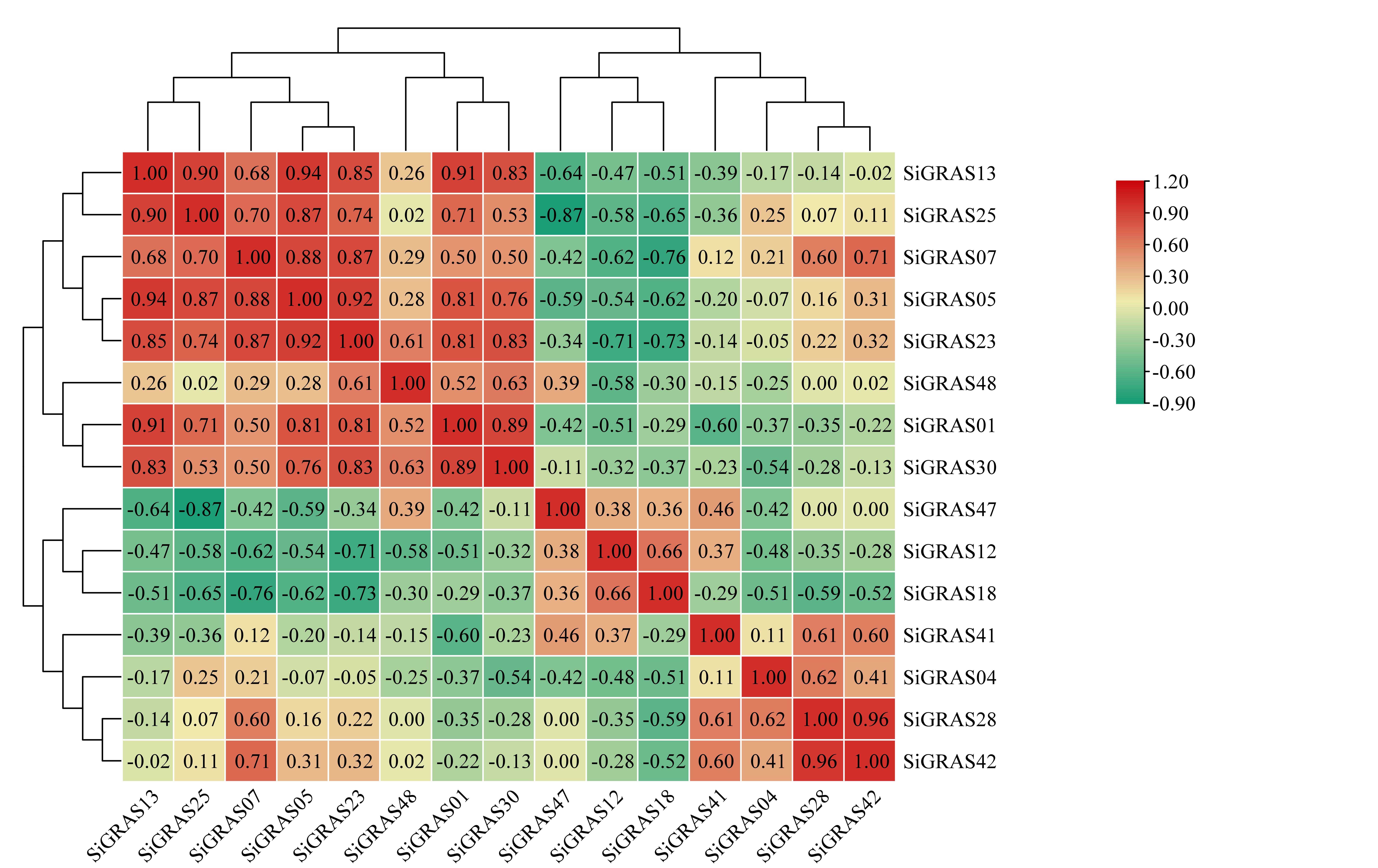

Supplement: Supplementary file 13 — Additional file 13 : Figure S4. The correlations 15 S. italica GRAS genes in several plant organs. Positive number: positively correlated; negative number: negatively correlated. The correlation coefficients of SiGRAS genes were defined by software Sigmaplot 12.0 based on Pearson correlation program. The correlation coefficient is defined as significant correlation with P-value lower than 0.05. [file 12870_2021_3277_MOESM13_ESM.jpg]

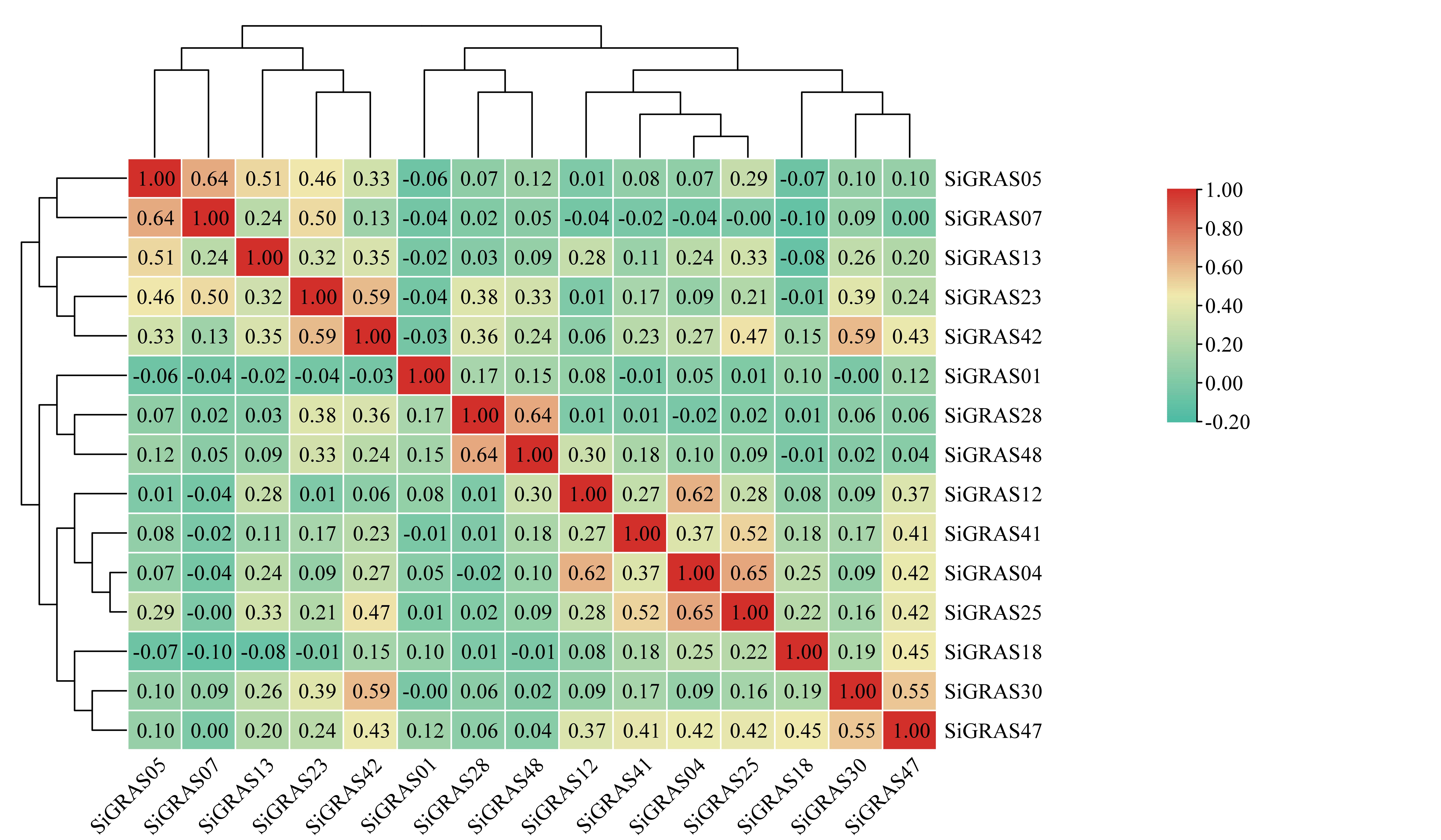

Supplement: Supplementary file 15 — Additional file 15 : Figure S6. The correlations 15S. italica GRAS genes in several abiotic stresses. [file 12870_2021_3277_MOESM15_ESM.jpg]
